# Book Reviews

**Published:** 1896-11

**Authors:** 


### BOOK REVIEWS.

Borderland Studies. Miscellaneous Addresses and Essays pertaining to Medicine and the Medical Profession, and their Relations to General Science and Thought. By George M. Gould, A.M., M.D.; formerly Editor of the *Medical News*. Blakiston, Philadelphia. For sale by J. F. Meegan, 23 Marietta street, Atlanta, Ga. Price, $2.

This is a book which contains some matter which is interesting especially to medical men, but none which is not suitable reading for the laity as well, while the major part of it deals with questions of supreme moment to every thoughtful mind. Such chapters as those on "Life and its Basis," "Immortality," "Character," "Maternal Love," etc., are very well worth careful reading. The theology of greatest interest for those whose lives are spent in touch with science, and especially in constant consideration of questions dealing with life and death, is that which discusses fundamental questions, the reasonableness of God, and of a future life, the individuality or otherwise of such life, the relationship of mind to brain, responsibility, and others of a like nature, more than questions concerning the trinity, the confessional, church government, which to our thinking take up more than their due share of attention, while the momentous questions are left too often to take care of themselves. It is with the former class that Dr. Gould's book in great part deals, and it does so in a manner which is decidedly attractive and in some parts original. We hardly think his argument conclusive concerning the necessary sinking of the individual life in a future state of existence into a common life, or that the two are not compatible in the future as they appear to be at the present. But entire agreement is not to be expected in the present state of knowledge. In spite of probable differences of opinion between the author and his readers, the book is highly to be commended as both instructive and pleasant reading.

DAVIS'S OBSTETRICS. A Treatise on Obstetrics. For Students and Practitioners. By Edward F. Davis, A.M., M.D., Professor of Obstetrics and Diseases of Infancy in the Philadelphia Polyclinic, Clinical Professor of Obstetrics in the Jefferson Medical College of Philadelphia. In one very handsome octavo volume of about 600 pages, with 217 engravings, and 30 full-page plates in colors and monochrome. Cloth, $5.00; leather, $6.00. Lea Brothers & Co., Publishers, Philadelphia and New York, 1896.

This book is divided into seven sections:—Pregnancy and Labor, Pathology of Labor, Obstetrical Operations, Abortion, Extra-uterine Pregnancy and the Puerperal State, Infancy in Health and Disease, Diseases of Infancy, and the Jurisprudence of Obstetrics. The author proves himself a man of high culture not only in the science of obstetrics, but also in the field of general biology. His work is concisely written, and contains an immense amount of information. The illustrations are a very conspicuous feature of the work. Besides being very numerous they are, on the whole, good, but we think it is possible to carry this element of the modern medical book a little too far. For instance, engravings of an ordinary binaural stethescope and of a tape measure, and photographs of such subjects as the obstetrician with his hand under a sheet, making a vaginal examination, the patient being practically invisible, can scarcely add to the usefulness of the work. Some of the photographs appear to be of small value, and are not over distinct. Wealth of illustration can, however, only be a fault on the right side. That errors in the text are not wholly wanting is evidenced by the fact that on page 61 Wolff's work on Epigenesis is referred to as having been undertaken in 1859 instead of 1759. These are comparatively small matters, and the book is one well worth study, and destined no doubt to be popular.

---

A TREATISE ON APPENDICITIS. By John B. Deaver, M.D., Surgeon to the German Hospital, Philadelphia. Containing 32 full-page plates and other illustrations. Published by Blakiston, Son & Co., Philadelphia, and on sale by J. F. Meegan, 23 Marietta street, Atlanta.

This is a beautifully printed and illustrated volume of 168 pages, in which the author gives the result of his experience with this now so famous disease. The chief point of discussion at the present time in connection with it is that of treatment, which is summed up in this book as removal of the appendix as soon as the inflammatory condition there is diagnosed, failing which one must rely mainly on properly administered purgatives, special stress being laid on the avoidance of opium. However true it may be that operation in experienced hands is in many cases the best procedure, it seems to us necessary that a word of warning should be given against promiscuous operation by those unaccustomed to such serious work, and we venture to think that in country practice the mortality will be greater by operative methods than where simple medical treatment is employed, even while we grant that some cases are practically certain to end in death without operation. The point is that this is work for the trained surgeon and not for the average general practitioner.

---

THE STUDENT'S MEDICAL DICTIONARY, including all the words and phrases generally used in medicine, with their proper pronunciation and definitions, based on recent medical literature. By George M. Gould, A.M., M.D. With elaborate tables of the bacilli, micrococci leucomains, ptomains, etc., of the arteries, ganglia, muscles, and nerves ; of weights and measures ; analyses of the waters of the mineral springs of the United States, etc. Tenth edition. Rewritten and enlarged. Published by Blakiston, Son & Co., Philadelphia, and for sale, $3.25, by J. F. Meegan, 23 Marietta street, Atlanta.

The author states in his preface that, despite their popularity, the older editions were unsatisfactory to him, and that the present volume is an entirely new one, and designed to take the place of the New Medical Dictionary and the Student's Medical Dictionary, the plates of which have been destroyed. To his larger work, the Illustrated Medical Dictionary, one is referred for more complete information.

The Student's Dictionary contains a sufficient outline of terms relating to medicine, and is so arranged and printed that reference is easy and satisfactory. We have noticed only one misprint, an accoucheuse being defined as a wife.

The book is highly to be commended and author and publisher to be congratulated on the appearance of the work.

---

A COMPEND OF GYNECOLOGY. By William H. Wells, M.D., Adjunct Professor of Obstetrics and Diseases of Infancy, in the Philadelphia Polyclinic; late Assistant Demonstrator of Clinical Obstetrics in the Jefferson Medical College, Philadelphia, etc. Blakiston, Philadelphia. On sale by J. F. Meegan, 23 Marietta street, Atlanta. Price 80 cents.

This is one of a class of books which are of doubtful value in preparing the student for a true knowledge of medical subjects, their only use being as an aid in cramming before an examination. But of its kind it is apparently one of the best, mainly on account of its numerous illustrations, which do more than any short description can to convey an understanding of the subjects under discussion. The matter is divided under headings in such a way as to recall information previously obtained, but is too condensed for first study. It is a good book of its kind.

---

A COMPEND OF DISEASES OF CHILDREN. Specially adapted for the use of Medical Students. By Marcus H. Hatfield, A.M., M.D., Professor of Diseases of Children, N. W. W. Medical School; Physician to Wesley Hospital, Home for Crippled Children, Chicago Orphan Asylum, etc. Second edition. Thoroughly revised, with a colored plate. Published by Blakiston, Philadelphia. On sale by J. F. Meegan, 23 Marietta street, Atlanta. Price 80 cents.

The author of this little cram-book or compend says that it is founded upon Dr. Ernst Kormann's "Compendium der Kinder-krankhuben," and that he has also drawn freely upon Henoch, Bouchut, Baginsky, Speinli, and others. It is concisely written, as was necessary for its purpose, which it fulfills well.

